# Emulgels as Fat-Replacing Systems in Biscuits Developed with Ternary Mixtures of Pea and Soy Protein Isolates and Gums

**DOI:** 10.3390/gels11070478

**Published:** 2025-06-20

**Authors:** Andreea Pușcaș, Anda Elena Tanislav, Andruța Elena Mureșan, Vlad Mureșan

**Affiliations:** Food Engineering Department, University of Agricultural Sciences and Veterinary Medicine, 400372 Cluj-Napoca, Romania; andreea.puscas@usamvcluj.ro (A.P.);

**Keywords:** protein–polysaccharide interactions, protein isolates, hydrogels, thermal denaturation, emulgels, fat replacer, biscuits

## Abstract

Hydrogels (Hy) were obtained with a ternary system of proteins (pea (P) or soy isolate (S) 2%), guar (0.5%), and xanthan gums (0.5%) and were subjected to thermal treatment (70 °C/20 min or 85 °C/15 min, or not) prior to structure formation. The FTIR spectra of the hydrogels and the turbidity test (spectrophotometrically red at 600 nm) were used for studying protein–polysaccharide interactions. Amplitude sweeps (0.01–100%) and flow behavior tests (0.1–100 s^−1^) were conducted for structure analysis. Emulgels were obtained by emulsification of the Hy with 40% or 60% sunflower oil. The centrifugal stability and texture (TPA test) of the emulgels were assessed and SND_40% exhibited the highest hardness (5.30 ± 0.23 N). Based on the results, SND_40%, PND_40%, SD70_40%, and PD_70% were chosen as fat-replacing systems in biscuit formulation. The textural, color, and stability attributes of the reformulated samples were compared with a reference containing margarine. Increased hardness and fracturability were determined for the emulgel-based biscuits, while the color parameters were statistically similar to the reference. Thermal treatments applied to enhance protein–polysaccharide interactions increased the structural performances of some emulgels, while their application as fat-replacing systems should be further evaluated since no statistical differences were recorded in the sensory evaluation of the reference and reformulated biscuits. Emulgels with tuned technological properties have the potential to replace saturated fats in foods.

## 1. Introduction

Emulgels are considered a biphasic system formed by a gelled phase and an unstructured organic phase or water. Recently, these structures were highly explored in the food engineering domain as drug delivery systems, because of their capabilities to retain and vehiculate both polar or unpolar compounds, providing enhanced stability and controlled release, transforming the products into functional foods [1]. There is a high possibility of designing edible emulgels; when hydrogels (Hy) are mixed with vegetable oils (O), Hy/O and O/Hy emulgels are obtained. Hydrogels with tuned technological and mechanical properties can be obtained if their fabrication is influenced by the pH, ions, temperature, or chemical or enzymatical crosslinking [2,3]. The impact of the external factors on the resulting emulgels has been more and more studied recently. Emulgels can be developed if water is mixed with oleogels (Og), Og/W or W/Og systems being developed in this case. Oleogels can be obtained in the direct or indirect path, leading to various structures.

Vegetal-sourced proteins, because of their composition, nutritional potential, and sustainable character, were already used for hydrogel and oleogel development and also in emulgel production, contributing to a clean label, which is highly demanded by consumers. Protein-based oleogels can be obtained by removing the water from Og/W or W/Og, with high internal-phase emulsions being obtained, or by solvent exchange procedures [4]. Soy and pea isolates were mainly used for developing such structures, the last presenting challenges during the dispersing phase. pH adjustments and supplementary dispersion with high-speed or two-step homogenizers were employed in the formation of an 8% aqueous protein solution [4]. Polysaccharides along with proteins produce hydrogels with improved properties, and enhanced strength and mechanical properties, possibly as a result of the “filling effect” or “packing effect” [5]. In terms of protein–polysaccharide interactions, previous studies followed the effect of the pH and polysaccharide concentration on the binary mixture of xanthan gum (XG) and micellar casein (MC) [6], the effect of ultrasounds on the functional and structural properties of whey protein isolate and gellan gum [7], or of dry or wet heating [8]. Ternary protein–protein–polysaccharide or protein–polysaccharide–polysaccharide hydrogels were also developed (ex: Xanthan-κ-carrageenan–egg yolk protein [9], Chitosan–wheat starch–whey protein) and the resulting structures presented compact strength, high gel strength, and high water-holding capacity [10]. These properties are desirable for emulgels, which can be obtained from hydrogels. A previous study investigated the interaction of xanthan gum (XG), enzyme-modified guar gum (EMG), and 2 wt% whey protein isolate (WPI) and the impact on the performance of an oil-in-water (O/W) emulsion containing 20% fish oil [11]. The structures formed by mixtures of proteins and polysaccharides have been recently evaluated in the reviews of Christophe Chassenieux and Taco Nicolai, 2024 [12] and Babu, Ashley, et al., 2024 [5]; thus, the application of such structures in food products is the next approach for stressing their usage on an industrial scale, to obtain reformulated products with improved nutritional status.

The properties of soy/pea protein hydrogels of various concentrations were already modulated to obtain effective xerogels, cryogels, and aerogels for food product development [13]. Emulgels containing 20% oil and hydrogels stabilized by protein–polysaccharide mixtures were already developed and characterized [11,14], but bakery products require higher fat contents in the formulation.

Guar gum can be used in food products at a max. amount of 2% and leads to changes in viscosity, its action being enhanced when it is combined with other gums [15]. Xanthan gum is also recommended to be used in mixtures, given its sourcing. The usage of both as emulsifiers is well known, while in baking, they can maintain the moisture and freshness of products [16]. The presence of proteins can influence the action of gums while proteins can form a gel-like network if they are thermally activated. Direct applications of hydrogels in foods, as a replacement for other ingredients, is still a challenge [17], for which reason the formation of emulgels could lead to food products with similar properties but improved nutritional/caloric content. Emulgel usage has already been explored in meat-based products (dry fermented foal sausages were obtained with alginate-based hydrogels mixed with sesame, tiger, and algal oil) [18] and in reduced-fat Bologna sausages (developed with olive oil emulgels based on chia mucilage and various gelling agents) [19]. The usage of emulgels as saturated fat replacers in frozen baked dough was also recently explored because of the potential of hydrocolloid action on ice crystals and improved structure [20]. Various oleogels have already been studied for replacing margarine, butter, or shortening in the formulation of biscuits [21,22,23,24], and fewer studies have been carried out to assess the suitability of emulsificated oleogels as fat-replacing systems in biscuits, despite the potential of obtaining low-fat products. 

Emulsion gels (77% water phase) enriched with β-glucans enabled a 50% replacement of margarine in biscuits, which presented comparable textural and microscopic properties with the reference [25]. Emulsions formed with 8% commercial fiber, 55% sunflower oil, and water led to a 50% replacement of palm oil in biscuits, with a high sensory acceptance being reported for the reformulated samples [26]. A total of 50% of butter was also replaced in shortbread cookie formulation with emulsion-filled gels, obtained from inulin and extra virgin olive oil; thus, achieving a 100% replacement of conventional fat, without altering the sensory attributes of biscuits, remains a challenge [27]. Bigels (50% hydrogel phase) were employed in manufacturing fat-reduced biscuits and the hardness of the reformulated samples was similar to the reference containing shortening, highlighting that the hydrogel type influences the physico-chemical characteristics [28].The aim of the current study was to evaluate the influence of thermal treatment on the structure of hydrogels obtained with ternary systems of proteins (2% pea or soy protein), guar gum (0.5%), and xanthan gum (0.5%), but also on the emulgels developed for 100% replacement of margarine in biscuits. The emulgels were obtained by the emulsification of hydrogels with 40% or 60% sunflower oil. In the current study we attempted to design effective emulgels, which would be structurally stable, with low adhesiveness and a compact structure.

The usage of emulgels in foods might bring obvious nutritional advantages and the current study will bring knowledge related to the feasibility of designing food products with such structures, in terms of structural performances and sensory acceptance.

## 2. Results

### 2.1. Turbidity of the Hydrogels

Since thermal denaturation can cause the unfolding of the proteins, aggregation, and rearrangement, determination of the turbidity in the spectrophotometric assay was employed to reveal the interaction of proteins with gums, as shown in Table 1.

Higher absorbance values of SD70 indicate that the 70 °C/20 min protocol could enhance the interaction of soy with polysaccharides. In the case of hydrogels with pea, which were subjected to thermal treatments (PD70, and PD85), the presence of larger aggregates was confirmed based on the results; thus, protein denaturation occurred and possible protein–hydrocolloid interactions [29]. Thermal treatment might be favorable for the pea protein-containing hydrogels. An absorbance of 0.10 was red for the pea protein solution prepared at 30 mg/mL (*w*/*v*), without the influence of any external factor, which was similar to what was recorded in our study [30]. The results are positive, since some studies suggest that ultra-sonication times can result in the formation of large protein aggregates [31]; thus, the applied ultrasound treatment did not negatively affect the hydrogels, but resulted in a positive outcome, that of improving protein solubility and the emulsification properties (as demonstrated by the centrifugal stability of the samples). The turbidity recorded for a black bean protein solution (10 mg/mL (*w*/*v*)) ranged between 0.4 and 0.6 [29]. If proteins alone would have been used as emulsifier agents, protein aggregates with smaller sizes would have been desired, since they could present higher emulsifying capacity.

### 2.2. FTIR Spectral Data Analysis

**Figure 1:**
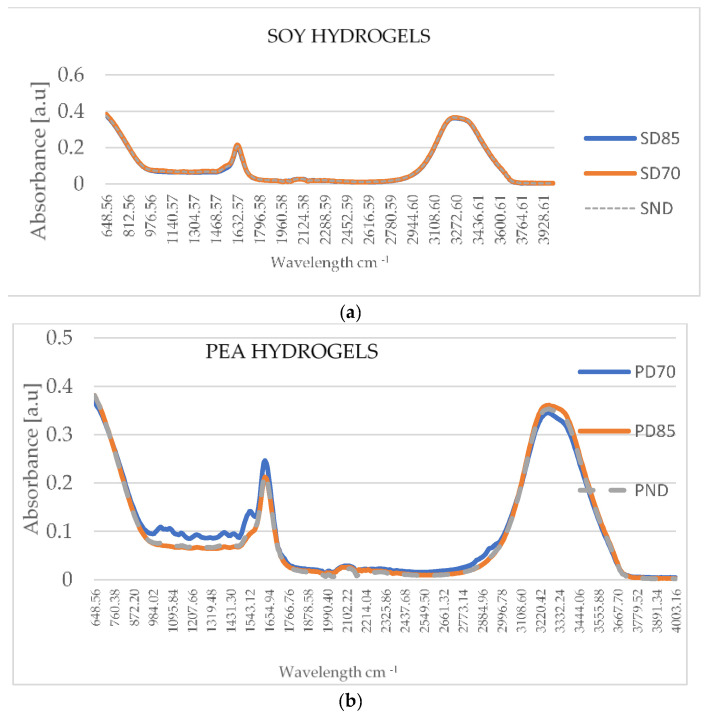
FTIR spectra of hydrogels developed from 3% ternary mixture of (**a**) soy protein isolate-xanthan-guar gum (**b**) pea protein isolate-xanthan-guar gum, subjected to thermal treatments (70 °C/20 min for PD70, SD70, 85 °C/15 min for PD85, SD85, or not SND, PND).

FTIR was employed as a powerful tool to determine the molecular and structural arrangements involved in the interaction of proteins and polysaccharides. After the protein was denatured by heat, its structure was refolded and assembled such that some hydrophobic groups were exposed, causing protein aggregation. Hydrophobic interactions, hydrogen bonds, and disulfide bonds are the main forces involved in protein aggregation and formed their characteristic peaks, as seen in the FTIR spectra (Figure 1). FTIR spectra of soy containing hydrogels, is presented in Figure 1a) and the O-H stretching resulted in the formation of peaks at 3220–3332 cm^−1^, representing either the stretching vibrations of hydroxyl (OH) groups present in the guar gum and xanthan gum, the OH of water, or the N-H bending vibrations of proteins. No shifts in or modification of the shape of the peaks occurred for soy Hy; thus, no major molecular modification occurred in this region. In the case of samples containing pea protein, higher absorbance values were recorded for PD85, explaining the higher bond in the structure (Figure 1b).

The peak in the 1654 cm^−1^ region can be assigned to the C=O stretching from the amide groups of proteins, this region being characteristic of the Amide I group, being a common marker for protein denaturation through thermal treatments. The peak formed at 1632 cm^−1^; in the spectra of SD70, higher values of absorbance (0.21 a.u.) were registered in comparison to those of SND and SD85 (0.20 a.u.). For the pea hydrogels, PD85 presented a higher absorbance than PND. PD70 presented a lower absorbance than PND and the peak presented a shoulder. The shoulder formed at 1558 cm^−1^, which was formed in the region where peaks appear due to the COO– and can be assigned to stretching vibrations of double bonds [32]. Polysaccharides are reported to stabilize proteins, reduce protein–protein interaction, and the polymeric network becomes mixed [33]. Changes in peaks at the 3600–3000 cm^−1^ region might be due to the interactions between the polysaccharides and proteins and this was visible for the pea Hy. Polysaccharides and proteins are reported to have non-covalent interactions [34], which typically can be detected when increases in the absorbance occur (PD85).

### 2.3. Hydrogel Rheological Properties

The linear viscoelastic (LVE) region represents the material’s resistance at rest or the elasticity under minimal deformation and was visible in a long range of strains (0.01–10%) for the hydrogels. The system’s responses were independent of the deformation magnitude and the structures were well maintained. The LVRs of the long length and high values of the elastic modulus (G’) depict the samples’ structural stability.

**Figure 2:**
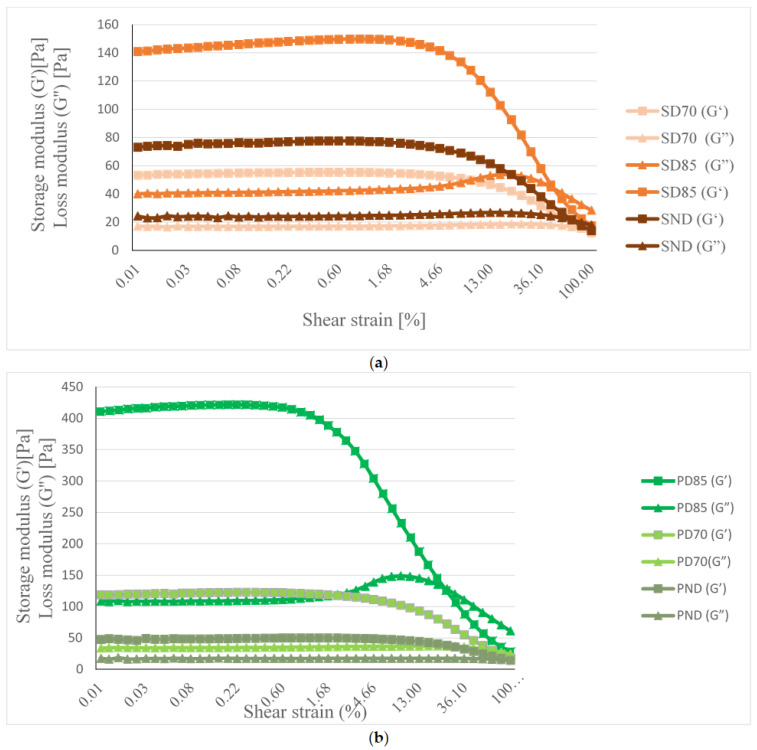
Storage modulus (G′) and loss modulus (G″) of hydrogels developed from 3% ternary mixture of (**a**) soy protein isolate–xanthan–guar gums and (**b**) pea protein isolate–xanthan–guar gum, as affected by shear strain (0.01–100%).

The storage modulus was higher for SD85 compared to SD70 and SND, as seen in Figure 2a). The values of the storage modulus of the pea hydrogels in the LVR region were higher than those of the soy-based samples, namely, the highest was 419.12 ± 59.73 Pa for PD85. For all the hydrogels, except PD85, the G″ values exhibited a constant plateau even at higher strain values.

The hydrogel developed with the ternary mixture containing PEA, which was thermally denatured, regardless of the time–temperature protocol, resulted in a stronger network compared to the pea-based sample, which was not subjected to thermal treatment (PND), or compared to the soy-based hydrogels (Figure 2b). However, SD85 and PD85 registered a G cross-over point, where G″ > G′ and their structures were collapsed, this aspect being of utmost importance in the next steps of the research. Pea hydrogels obtained by pH shift methods were reported to create weak hydrogels even at concentrations up to 15% [35]; thus, the protein–polysaccharide mixtures are efficient in creating strong structures. The addition of 0.5% xanthan and 0.5% guar gum to the pea isolate and their dissolution using ultrasounds contributed to the formation of strong hydrogels. Modified pH shift methods also led to increased gel mechanical strength (21 kPa) and presented high water retention (95%) [1]. In another study, 20% pea hydrogel registered a G′ of 13.64 ± 0.24 × 10^2^ (Pa), and it was concluded that increases in the protein concentration might positively affect the rheological parameters [14].

Polysaccharides are responsible for controlling the rheological properties, including the viscosity of the aqueous phase [36]. The effect of the thermal treatments on the interactions between the polysaccharides and proteins created the following effects on the viscosity of the samples: at the beginning of the analysis, SD70 presented a viscosity of 159,565.00 ± 321.73 mPa ×s and SD85 had a similar value of 159,780.00 ± 44,519.30 mPa × s, but at higher shear rates, the viscosity of SD70 became considerably lower than that of SD85.

**Figure 3:**
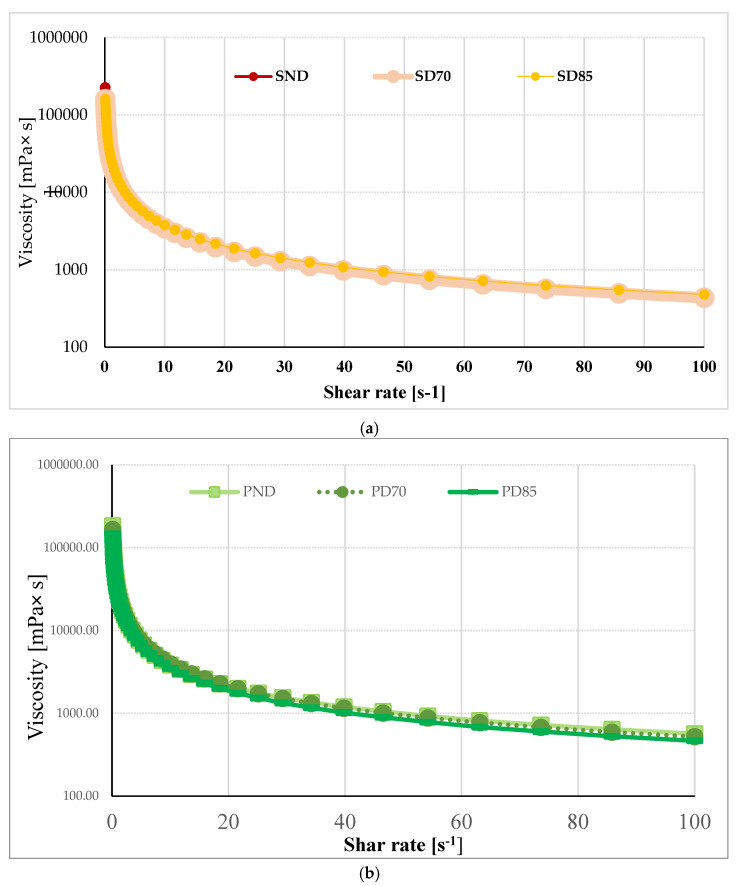
Flow curves of hydrogels developed from 3% ternary mixture of (**a**) soy protein isolate–xanthan–guar gums and (**b**) pea protein isolate-xanthan-guar gum, as affected by shear rate (0.1–100 s^−1^).

As seen in Figure 3, the samples exhibited shear-thinning behavior; the viscosity of SND decreased from 225,520.00 ± 77,173.60 mPa × s to 483.00 ± 4.12 mPa × s when the shear rate was increased to 100 s^−1^. At low shear rates, SND presented higher values of viscosity, followed by SD70 and SD85, while in the entire share rate domain, for the pea-based hydrogels, a similar hierarchy was obtained, with PND exhibiting the highest values (Figure 3). However, the pea-based hydrogels presented lower viscosity values (184,460 ± 7707.46 mPa × S for PND) at the beginning of the experiment compared to the soy-based hydrogels.

It was reported that the presence of a non-adsorbing polysaccharide (xanthan) in the continuous phase can lead to the formation of strong networks. In emulsification, a high viscosity is desired for the continuous phase, because the particles cannot move in such media and the sample will not destabilize, avoiding one of the factors that shortens emulsions’ shelf life: creaming.

In another study, unheated 2% *w*/*w* soy protein isolate suspension at pH 2.0 or a thermally treated sample (at 80 °C for 4 h) exhibited Newtonian flow and lower viscosity (around 1 mPa × s), meaning that gums are able to increase the viscosity of protein-based hydrogels in a10^5^ magnitude [37].

### 2.4. Emulgel Textural Analysis

**Figure 4:**
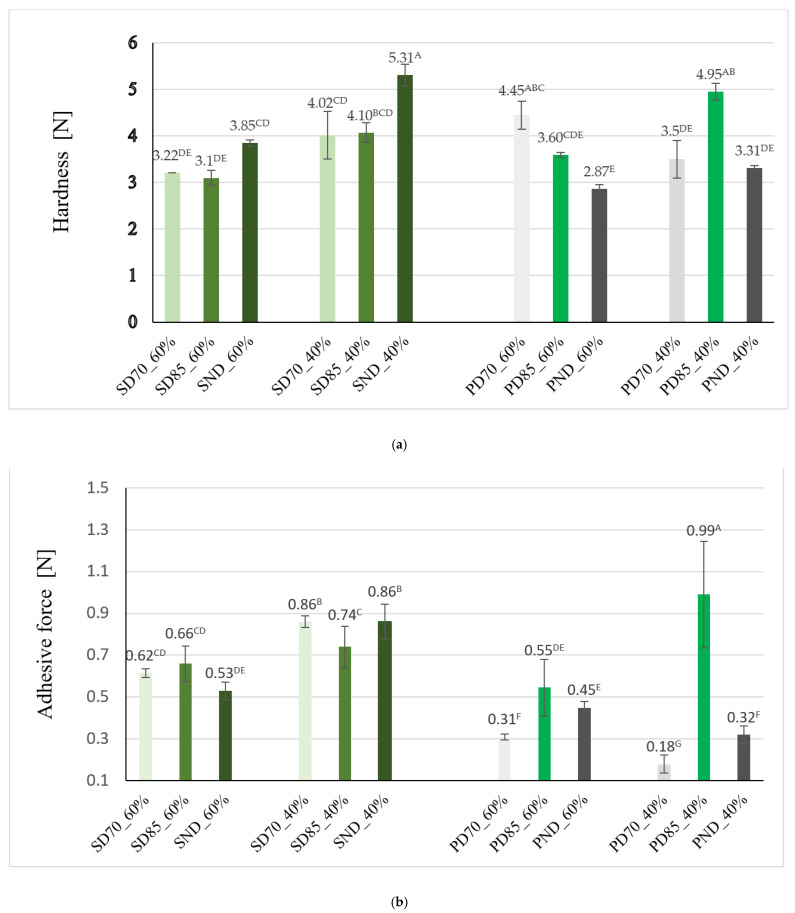

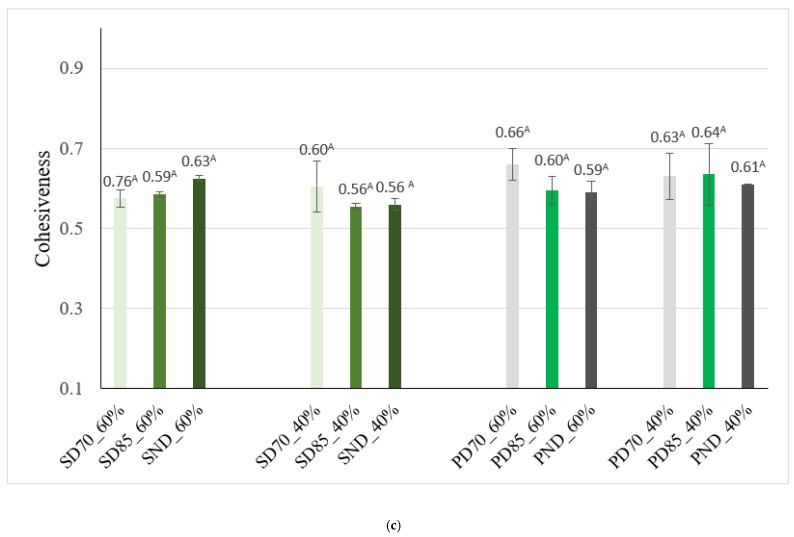
Textural parameters: hardness (**a**), adhesive force (**b**), and consistency (**c**) of emulgels developed from the Hy and 40% or 60% sunflower oil. Identical superscript letters indicate no significant difference (*p* > 0.05).

TPA is a powerful tool for the examination of the interactions between the ingredients of a product [38]. Thermal treatment of the hydrogel decreased the values of hardness for the soy-based emulgels, while for the pea-based samples, it increased the hardness, as seen in Figure 4a). The highest hardness was registered for SND_40% (5.30 ± 0.23 N/540.95 g after conversion) and SND_60%, while PND_40% also presented higher values of hardness than PND_60%.

The thermal treatment positively influenced the hardness of the pea emulgels, since a higher hardness was obtained for PD85_40% and PND70_40% compared to PND_40%. PD85_60% and PND70_60% also registered higher values of hardness compared to PND_60%. Butter at room temperature (24 °C ± 1) was reported to exhibit a hardness value of 241.74 ± 61.40 g [22].; thus, with thermal treatment, tuned properties of emulgels can be achieved to meet the desired values of the textural attributes of conventional fats.

36 types of gelled emulsion were prepared from 40% extra virgin olive oil (EVOO) and different hydrocolloids (alginate, cellulose, collagen, or k-carrageenan), in order to be studied as plant-derived shortening replacers. The samples registered hardness values ranging between 193.67 and 332.43 g [22]. Similar values were obtained for the emulsions with 40% oil content in our study, for which the hardness ranged between 292.65 and 392.59 g (after conversion from N), except for the PD70_60% sample. PD70_60% registered the highest hardness among the emulgels containing 40% oil, namely 453.77 g.

For PD85_40%, despite the superior hardness achieved, increased values of adhesive force were also determined, for which reason it is less desired to use this sample in the formulation of food products. The adhesive force of PD70_40% was the lowest, accounting for 0.18 ± 0.04 N, followed by that of PD70_60% and PND_40%, as seen in Figure 4b. Except for PD85_40%, emulgels manufactured with the pea isolate presented lower adhesive force than samples with soy protein in the formulation. Thus, the application of the 70 °C-20 min thermal protocol in obtaining samples contributed to emulgels with better adhesive attributes. The adhesive force of the soy-containing emulgels varied between 0.53 and 0.86 N and was influenced by the oil fraction in the emulgel, being lower in the samples with 40% oil in the composition.

Cohesiveness is a mechanical textural parameter that can provide information about the interconnection of some particles [39] and about the restructuring ability of the system after successive shear forces are applied [38]. The high cohesiveness values highlight the good internal organization and mechanical performances of the samples. The emulgels that displayed improved cohesiveness were PD70_60%, PD70_40%, PND_40%, and SND_60%. On the contrary, the soy-based emulgels with 40% oil fraction presented slightly higher cohesiveness, along with SD70_40%, but the values were not statistically significant.

### 2.5. Biscuit Properties

In soy hydrogel fabrication, the 70 °C/20 min thermal protocol enhanced the interaction of soy with polysaccharides and led to changes in the Amide I groups, as revealed by FTIR analysis. For the pea-based samples, the 70 °C/20 min protocol also led to changes in the turbidity values and to higher absorbance in the 1640 cm^−1^ region of the FTIR data. Samples that were subjected to the 85 °C/15 min thermal protocol exhibited a G cross-over point at lower shear strains, meaning their gel structure would be destroyed during deformation. SND and PND exhibited higher values of viscosity at the beginning of the experiment; thus, it can be considered a fat-replacing system. SND_40% presented the highest value of hardness, which is also an important aspect when considering a fat-replacing system. For these reasons, SND_40%, SD70_40%, and the counter pea-based samples were taken into consideration for biscuit formulation. When emulsifiers like proteins are present in the batter, they could incorporate more air bubbles into it, aeration being of utmost importance for the texture of the biscuits. Biscuits with emulgels as fats exhibited higher values of hardness in comparison to the reference, and this is a current disadvantage that was also reported in previous studies regarding reformulated biscuits [40]. The hardness of some cookies elaborated in another study with emulgels (40 % oil phase), a hydrophobic surfactant (PS 80, 0.12 %), and alginate was of almost 50 N (5098.58 g after conversion), this high value being attributed because of a lower fat content and its replacement with a 60% water phase, while that of their control cookie was almost two times lower [40]. In the current study, emulgels containing 60% oil were employed in the manufacturing process, to obtain the textural and sensory attributes that fats might confer.

The hardness of biscuits with SND70_40% and PND70_40% was higher (Table 2). However, the hardness of all of the emulgel-based samples was higher than that of the reference containing margarine, which registered a hardness of 180 ± 11.31 g.

Fracturability, the force necessary to break a biscuit’s structure, was the highest for the biscuit manufactured with SND70_40% and that of the reference was almost 10 times lower. Previous studies report increased fracturability of biscuits with conventional fat replaced [21].

A higher value of the L* parameter (brightness) indicates that the Maillard reactions during baking were hindered; B_SND70_40% presented the lowest value for this parameter, namely 55.19 ± 1.43, which was statistically similar to the reference. The rest of the samples registered higher L* values. Despite the similarity in L*, B_SND70_40% registered the highest value for ∆E, the parameter that measures if the color differences can be perceived by consumers. However, the values registered for ∆E, ranging between 3.36 and 5.49, were not statistically different from each other. Also, B_SND70_40% registered a statistically higher value than the rest of the samples for the a* parameter, the shade of redness (+a). The reference with margarine had an a* of 12.28 ± 0.57, while the rest of the emulgel-based biscuits registered values between 12.67 and 13.76. In terms of b*, the shade of yellowness, no statistically significant differences were determined.

Fat migration of the freshly baked biscuits varied between 0.19 and 0.22%. Similar values were reported for biscuits prepared with 5% beeswax oleogel, the oil loss remaining a challenge to be solved in emulgel-based biscuits [21]. For the biscuit with the emulgel containing denatured pea protein, lower values were obtained, but not statistically significant. Lower values were obtained for biscuits similar in composition to our samples, but they were formulated with 2–3% Candelilla wax-based oleogels as fat, the fat phase representing 24% of the dough composition [21].

### 2.6. Results Regarding Sensory Analysis

**Figure 5:**
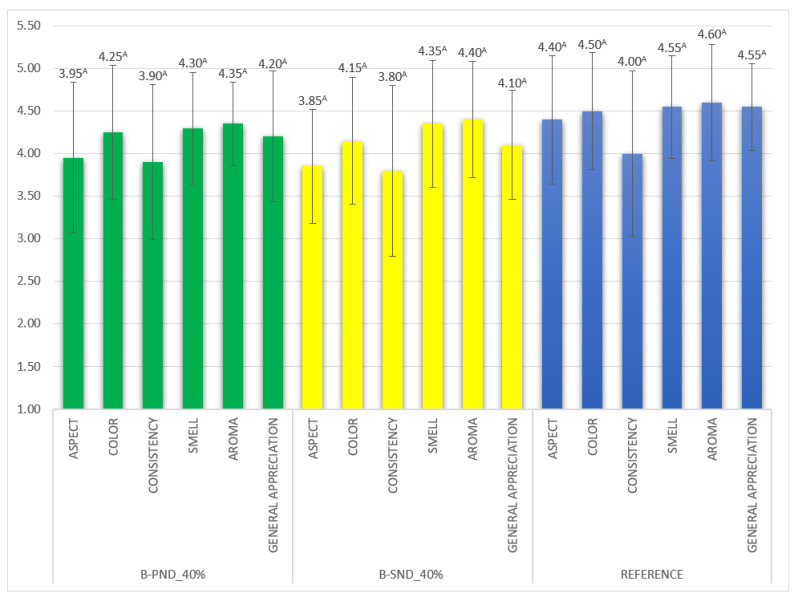
Sensory acceptability of biscuits evaluated in hedonic test. Identical superscript letters indicate no significant difference (*p* > 0.05).

The hedonic sensory scores of the reformulated biscuits in terms of smell and taste were statistically similar to those of the reference, as seen in Figure 5. The aspect of the samples, which were scored lower than the reference, led to some differences, but not because of the color of the samples, since in the instrumental analysis, the color parameters were similar to those of the reference. The aspect of the biscuits with PND_40% was scored with 3.95 ± 1.00 while that of SND_40% with 3.85 ± 0.85. Consistency also received lower scores for the reformulated biscuits.

Previous results report that when fat is replaced in biscuits, the taste/smell, which create the flavor of the sample, might be affected and evaluated with lower scores in sensory analysis [41], while the emulgel-based biscuits received high scores both for the smell and taste, comparable with the reference, as seen in Figure 5.

The hedonic evaluation results showed that the differences between the control and reformulated products were low, no statistical differences being registered.

A previous study reports that cake formulated with emulgel obtained from high oleic sunflower oil (20%) and an isolated soy protein suspension was scored much lower in the hedonic test for the color parameter and reformulated products might not have the same intensity of color as the classic product [42]. Except for the flavor, the reformulated cakes received lower scores for each parameter.

## 3. Conclusions

The type of protein isolate (pea or soy), despite its low amount used in the composition of the hydrogel, along with the time–temperature protocol explored to promote the polysaccharide–protein interactions prior to hydrogel formation, influenced the performance of the emulgels. FTIR revealed that thermal treatment promoted the protein–polysaccharide interactions, and their non-covalent interactions led to increased absorbance values for PD85 and PD70 in the 1632–1640 cm^−1^ region or in the 3300 cm^−1^ region. A turbidity test confirmed that for SD70 and PD85, larger aggregates were formed because of the protein–hydrocolloids interactions.

For the hydrogels developed with the ternary mixtures containing pea proteins, thermal denaturation led to the formation of stronger gels. Samples with soy formed weaker gels, but with higher stability; their G_LVR_ was determined at a shear strain of 0.01 and no G cross-over point (where G″ > G′) was detected. At low shear rates, the SND hydrogel presented higher values of viscosity, followed by SD70 and SD85, while in the entire share rate domain, for the pea-based hydrogels, a similar hierarchy was obtained. Thermal treatment influenced the hydrogels’ viscosity. At higher shear rates, SD85 had higher viscosity than SD70, while PD85 had lower viscosity than PD70 in the whole shear rate range.

The emulgels’ textural attributes were influenced by the oil content in the formulation and the thermal treatment of the hydrogel. PD85_40% and PD85_60% presented higher values of hardness than the rest of the pea-containing emulgels, but also a high adhesive force, which is less desired. PD70_40% registered the lowest value of adhesive force, transforming the emulgel into a candidate for replacing margarine in biscuits. The application of the 70 °C-20 min thermal protocol contributed to samples with better adhesive attributes.

Based on these results, biscuits were formulated with SD70_40%, SND_40%, PD70_40%, and PND_40%. Biscuits with emulgels as fat exhibited higher values of hardness and fracturability in comparison to the reference. However, B_SND_40% and B_PND_40% presented better textural attributes and were tested for consumers’ preferences in a hedonic test along with the reference. No statistically significant differences were obtained for the reformulated samples in comparison to the reference.

The reformulated biscuits scored well for the taste and smell, but consistency, the textural attribute, scored low grades (3.80–4.00), both for the reformulated biscuits and the reference. The instrumental analysis of color revealed that ∆E, the parameter that measures the color differences between the reformulated samples and the reference, was not statistically different for any of the samples; however, the aspect, color, and overall appreciation of the B_SND_40% received lower scores. Further improvements are required to improve the textural attributes and oil leakage of the samples, while hydrogels and emulgels with tuned properties due to thermal treatments can be explored as fat-replacing systems in other food products.

## 4. Material and Methods

### 4.1. Materials

Soy and pea isolates, xanthan gum, and guar gum were offered by Supremia, Romania. Sunflower seed oil, flour, ammonia, baking powder, and sugar were purchased from a local market.

### 4.2. Samples Preparation

#### 4.2.1. Ultrasound-Assisted Hydrogel Formation

In order to obtain hydrogels, 2% protein powder, soy (S) and pea isolate (P), 0.5% guar gum, and 0.5% xanthan gum were weighed and dispersed into water, the mixture being subjected to ultrasound treatment (JULABO GMBh, Seelbach, Germany), for 1 h (100%, 25 °C) for better dispersion. The hydrogels were subjected to thermal treatments, which aimed to functionalize the proteins within the composition, namely they were heated in the oven at 85 °C for 15 min, or at 70 °C for 20 min, or not heated at all. Then, the samples were stored overnight at 4 °C in order to ensure hydration of the hydrocolloids and the hydrogel formation. Samples’ codification based on their composition and thermal treatments are summarized in Table 3.

#### 4.2.2. Emulgel Formation

Prior to emulsification at 13,500 rpm (8 min.), the oil was heated at 60 °C (Memmert UF55 Oven, Germany), and mixed with 40% or 60% hydrogel, the resulting samples’ codification being presented in Table 4; the amount of hydrogel used in formulation was included in the sample codification.

### 4.3. Obtaining Biscuits

In order to obtain the nutritionally improved biscuits, SND_40%, SD70%, PND_40%, and PD70_40% emulgels replaced the margarine in the recipe of the biscuits. Emulgels were mixed with sugar (18.83%), then with the solid ingredients (flour-43.65%, ammonia, baking powder, cocoa powder (0.63%,), the dough being modeled to obtain round-shaped biscuits. Chocolate chips were also added. Biscuits were baked at 200 °C for 15 min in the oven, then cooled to room temperature.

### 4.4. Characterization of Hydrogels

The turbidity of hydrogel solutions (10 mg/mL deionized water) was evaluated, with the value of spectrophotometric absorbance red at 600 nm using a UV-VIS 1700 Shimadzu spectrophotometer (Kyoto, Japan) [18]. This was conducted in order to study the protein–polysaccharide interaction as a result of thermal denaturation. Distilled water was used as a blank.

The FTIR spectra of hydrogels (acquired with an Agilent Cary630 (Agilent Technologies, Chelmsford, MA, USA) equipped with ATR Diamond sampling module, 600–4000 cm^−1^) were determined, to observe the molecular arrangements in hydrogels.

The hydrogel rheological properties (measured at 25 °C) were studied using the Anthon Paar MCR 302 (Anton Paar, Graz, Austria) rheometer equipped with a Peltier system and water bath (Julabo, Seelbach, Germany) for temperature control. For amplitude sweep, PP25 geometry was used and the shear strain was varied from 0.01 to 100%, while to study the flow behavior, the samples were subjected to shear rates between 0.1 and 100 s^−1^.

### 4.5. Emulgels’ Structural Characterization

Emulgels were centrifuged at 3000 rot/min (1107 g force after conversion) for 30 min with DLAB Centrifuge (DLAB Scientific Co., Beijing, China) and 9000 rot/min (9961 g force- after conversion) for 15 min using HETTICH UNIVERSAL 320 R Centrifuge (Andreas Hettich GmbH & Co. KG, Tuttlingen, Germany) to determine their stability. Centrifugation was performed at room temperature. No water or oil separation was observed.

The texture of the emulgels was assessed by a TPA test, using a CT3 Brookfield Texture Analyzer (Brookfield Engineering Labs, Middleboro, MA, USA), TA25/1000 geometry, with a 50% compression of the samples. The hardness, adhesive force, and cohesiveness of the samples were given by the software of the device.

### 4.6. Biscuits’ Characterization

#### 4.6.1. Textural, Color, and Stability Attributes

A compression (50%) test using TA7 geometry was performed to assess the hardness and fracturability of the biscuits, using a CT3 Brookfield texture analyzer (Brookfield Engineering Labs, Middleboro, MA, USA). L*, a*, b* color parameters were assessed with an NR200 (3NH, Shenzhen, China) portable colorimeter. Oil loss was determined by weighing the oil absorbed by a filter paper on which the samples were stored for 72 h.

#### 4.6.2. Sensory Analysis

**Figure 6:**
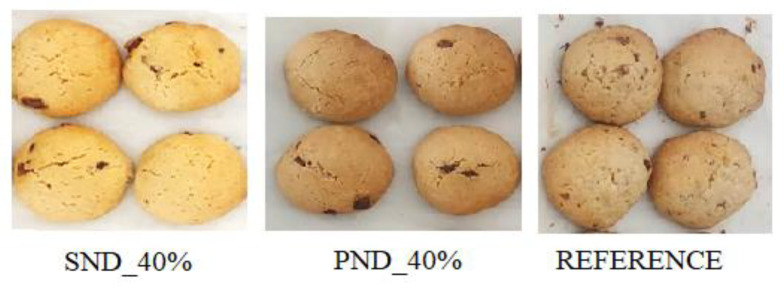
Appearance of the reference biscuits or samples obtained with emulgels (60% oil and 40% hydrogel obtained with 2% pea (PND_40%) or soy protein (SND_40%), 0.5% guar gum, and 0.5% xanthan gum, which was not subjected to thermal treatment).

A hedonic test related to 5 points was performed with 20 naive panelists, in order to evaluate the preferences of consumers regarding the reformulated biscuits. Hydrogels that were not subjected to thermal denaturation were used for emulsification with 60% oil and biscuit formulation. Samples were coded and anonymized; AEJ was the biscuit formulated with PND_40% emulgel, ZRV the biscuit formulated with SND_40% emulgel, and XYZ a reference formulated with margarine, which are represented in Figure 6. The evaluated attributes were color, consistency, smell, taste, and overall appreciation.

### 4.7. Statistical Analysis

Statistical analysis was performed in MiniTab Software 21.4.0. Two-way ANOVA was performed for turbidity and one-way ANOVA for biscuits’ properties, followed by Tukey comparisons test at a significance level of *p* < 0.05. All results were presented as mean ± SD (standard deviation). At least two replicates were performed for the samples.

## Figures and Tables

**Table 1:** The value of absorbance red at 600 nm for the hydrogel solutions (10 mg/mL (*w*/*v*)).

Samples	Absorbance 600 nm	Samples	Absorbance 600 nm
SND	0.15 ± 0.00 ^Bb^	PND	0.09 ± 0.00 ^Ac^
SD70	0.17 ± 0.07 ^Ba^	PD70	0.22 ± 0.07 ^Ab^
SD85	0.13 ± 0.00 ^Bc^	PD85	0.24 ± 0.01 ^Aa^

SND = hydrogels structured with 2% soy and gums, not subjected to thermal treatment, SD70 = hydrogels structured with 2% soy and gums, subjected to thermal treatment (70 °C/20 min), SD85 = hydrogels structured with 2% soy and gums, subjected to thermal treatment (85 °C/15 min). PND = hydrogels structured with 2% pea and gums, not subjected to thermal treatment, PD70 = hydrogels structured with 2% pea and gums, subjected to thermal treatment (70 °C/20 min), PD85 = hydrogels structured with 2% pea and gums, subjected to thermal treatment (85 °C/15 min). Capital letters indicate there was a statistically significant difference between groups, while small identical superscript letters indicate no significant difference (*p* > 0.05).

**Table 2:** Characterization of the biscuits obtained from emulgels developed with 40% hydrogel.

Biscuit Sample	Hardness [g]	Fracturability [g]	L*	a*	b*	ΔE	Oil Loss (%)
B_SND_40%	371 ± 31.11 ^a^	260 ± 48.08 ^a^	58.66 ± 0.85 ^a b^	13.76 ± 2.16 ^b^	30.77 ± 3.11 ^a^	4.05 ± 0.56 ^a^	0.22 ± 0.0 ^a^
B_SD70_40%	612 ± 55.15 ^a^	350 ± 50.00 ^a^	55.19 ± 1.43 ^c^	17.35 ± 0.66 ^a^	30.69 ± 0.48 ^a^	5.49 ± 1.19 ^a^	0.22 ± 0.0 ^a^
B_PND_40%	310 ± 65.05 ^a^	216.5 ± 16.26 ^a^	59.61 ± 0.11 ^a^	12.99 ± 1.33 ^b^	31.73 ± 1.91 ^a^	3.53 ± 0.85 ^a^	0.21 ± 0/01 ^a^
B_PD70_40%	338.5 ± 24.74 ^a^	231 ± 22.62 ^a^	59.65 ± 0.81 ^a^	12.67 ± 0.53 ^b^	30.87 ± 0.59 ^a^	3.36 ± 0.78 ^a^	0.19 ± 0.01 ^a^
Reference	180 ± 11.31 ^b^	39 ± 7.07 ^b^	56.34 ± 0.70 ^bc^	12.28 ± 0.57 ^b^	30.87 ± 0.59 ^a^	-	-

Identical superscript letters indicate no significant difference (*p* > 0.05).

**Table 3:** Hydrogel Samples’ codification based on their composition and thermal treatment.

Hydrogel Codification (97% Water) Based on the Ternary Mixture Composition/THERMAL Treatment	Denaturated at 85 °C for 15 min	Denaturated at 70 °C for 20 min	Not Denaturated
2% pea isolate, 0.5% guar gum, 0.5% xanthan gum.	PD85	PD70	PND
2% soy isolate, 0.5% guar gum, 0.5% xanthan gum.	SD85	SD70	SND

**Table 4:** Emulgel Samples’ codification based on their composition and thermal treatment.

Emulgel Codification Composition/Thermal Treatment	Pea Protein + 40%OIL	Pea Protein + 60%OIL	Soy Protein + 40%OIL	Soy Protein + 60%OIL
70 °C for 20 min	P70_60%	P70_40%	S70_60%	S70_40%
85 °C for 15 min	P85_60%	PND85_40%	S85_60%	S85_40%
No thermal treatment	PND_60%	PND_40%	SND_60%	SND40%

## Data Availability

The original contributions presented in this study are included in the article. Further inquiries can be directed to the corresponding author.
